# Nutrient deprivation increases CD3 expression in RAW cells and augments the CD3-induced proinflammatory profile, associated with NFAT and IRF-1

**DOI:** 10.3389/fimmu.2026.1857007

**Published:** 2026-07-21

**Authors:** Ranferi Ocaña-Guzman, Lucero A. Ramon-Luing, Jahir Mendoza-Ruiz, Guillermo López-Chávez, Alondra Hernández-Hernández, A. Yarelli Huerta-Zarco, Julio Flores-Gonzalez, Leslie Chavez-Galan

**Affiliations:** Laboratory of Integrative Immunology, Instituto Nacional de Enfermedades Respiratorias Ismael Cosio Villegas, Mexico City, Mexico

**Keywords:** CD3 expression, macrophage activation, nutrient deprivation, proinflammatory, RAW 264.7 macrophages

## Abstract

**Background:**

Myeloid cells can express lymphoid markers, including components of the CD3-TCR complex. CD3+ macrophages have been identified in both infectious and non-infectious pathologies; however, their origin and signaling mechanisms remain poorly studied, primarily due to their low prevalence in human samples. Utilizing the RAW murine macrophage cell line as a model, this study aimed to ascertain whether nutrient deprivation induces CD3 expression and to identify the signaling molecules involved in the activation of a CD3-dependent proinflammatory profile.

**Methods:**

Firstly, the impact of environmental stress, specifically nutrient deprivation, on CD3 expression over a period was assessed. Subsequently, RAW cells were stimulated with anti-CD3 and IgG2a, and the profile of proinflammatory cytokines was evaluated alongside the expression of signaling proteins, including NFAT, c-Jun, and IKK. Furthermore, the transcriptional regulation of *IRF-1*, *GATA-3*, and *MAFB* was examined. Lastly, the functional capacity of CD3+ RAW cells was determined through a phagocytosis assay.

**Results:**

RAW cells were found to express molecules classically associated with T cells, including CD3, TCR, and CD4. Notably, CD3 expression was significantly upregulated at both the protein and transcriptional levels under nutrient deprivation, although it did not reach the levels observed in T cells. Functionally, CD3+ RAW cells exhibited enhanced phagocytic activity toward latex beads. Furthermore, stimulation with anti-CD3 plus IgG2a induced a robust proinflammatory cytokine response, characterized by the secretion of IFN-γ, TNF, and IL-6 as early as 5 hours post-stimulation. This response was associated with activation of signaling pathways involving NFAT, c-Jun, and IKK, along with increased IRF-1 expression and downregulation of MAFB, supporting the establishment of a CD3-dependent proinflammatory profile in RAW cells.

**Conclusion:**

Our findings establish RAW macrophages as a model to study CD3+ signaling machinery in myeloid cells. We demonstrate for the first time that nutrient deprivation induces CD3 expression and that the resulting CD3-driven proinflammatory program is associated, at least in part, with NFAT and *IRF-1*. These conclusions provide new insights into the origin and functional significance of CD3+ macrophages and offer a valuable platform for investigating the impact of this pathway on innate immune responses across various pathological contexts.

## 1 Introduction

In recent decades, unconventional myeloid cell subpopulations, including neutrophils, eosinophils, monocytes, and macrophages, have been identified to express receptors typically associated with lymphoid cells, such as the CD3-TCR complex (1). These subpopulations have been investigated in various pathological contexts, including tumors and atherosclerosis; however, several questions regarding their origins and functional roles remain poorly understood (2, 3). Moreover, the occurrence of monocytes and macrophages expressing the TCR-αβ receptor has been documented during *Mycobacterium tuberculosis* (Mtb) infection. Notably, TCR expression in these macrophages decreases following anti-tumor necrosis factor (TNF) treatment, underscoring the significance of TNF in the development of this cellular subset (4).

In a murine model of *Mycobacterium bovis* strain Bacillus Calmette-Guérin (BCG) infection, an increased population of CD3+ macrophages was observed at sites of infection (5). This population comprises three subsets: CD3+TCR- macrophages, CD3+TCR-αβ+ cells, and a smaller proportion of CD3+TCR-γδ+ cells. Each subset exhibits distinct phenotypic and functional characteristics, including CD1 molecule expression and CD3-dependent pro-inflammatory cytokine secretion (5, 6). A previous study demonstrated that tuberculosis (TB) patients, regardless of first-line drug sensitivity or resistance, exhibit higher frequencies of CD3+ monocytes than healthy donors (7). Following six months of anti-TB therapy, patients with drug-sensitive TB showed monocyte frequency normalization relative to the control group. In contrast, those with drug-resistant strains achieved normalization only after eighteen months of treatment.

Subsequent investigations employing an *in vitro* model of human monocyte-derived macrophages demonstrated that infection with the attenuated H37Ra strain did not modify the prevalence of CD3+ cells. In contrast, infection with the virulent H37Rv strain increased CD3+ TCR+ macrophage populations (8). The exploration of these rare populations in human subjects is limited, primarily due to their low abundance. Nonetheless, using the RAW 264.7 murine macrophage cell line (hereafter referred to as RAW cells), it was found that a substantial proportion of RAW cells are CD3+ (>50%), thereby establishing this model as a valuable tool for further investigation (9).

More recently, Antonopolonou T. et al. demonstrated that RAW cells express additional T lymphocyte receptors, including TCR-αβ, TCR-γδ, CD4, and CD8, alongside low CD3 levels. Interestingly, stimulation of FcγR-II/III receptors reduced TCR expression, suggesting an alternative mechanism for TCR membrane translocation. Furthermore, stimulation of these cells with antigens and a recombinant IgG-2α fragment targeting FcγR-II/III induced IL-2 production (10).

The current evidence indicates that macrophages can express components of the CD3/TCR machinery and respond to CD3-associated stimulation. Nonetheless, it remains unclear which environmental conditions promote CD3 expression in macrophages and which signaling pathways underlie the subsequent inflammatory response. In this study, we employed the RAW cell line to investigate whether nutrient deprivation enhances CD3 expression and to characterize the signaling pathways linked to CD3-induced proinflammatory activation. Additionally, we assess the potential of this model for mechanistic studies of CD3-positive myeloid cells, which are challenging to isolate in sufficient quantities from primary human samples.

## 2 Methods

### 2.1 Ethics statement

This study was approved by the Institutional Ethics Committee of the Instituto Nacional de Enfermedades Respiratorias Ismael Cosío Villegas (Protocol number B26-23) in Mexico City. All procedures were carried out in accordance with the 1964 Helsinki Declaration and the ethical standards of the Institutional Ethics Committees.

### 2.2 RAW cell culture

The RAW cell line was obtained from the American Type Culture Collection (ATCC). Upon thawing, cells were cultured at a concentration of 1 × 10^6^ cells in a TC-25 flask (Costar, ON, Canada) using RPMI 1640 medium supplemented with 10% fetal bovine serum (FBS; GIBCO, Grand Island, NY, USA), 100 units/mL of penicillin, 10 µg/mL of streptomycin, and 250 ng/mL of amphotericin B (Sigma-Aldrich, St. Louis, MO, USA). After 48 hours of incubation at 37 °C in a humidified atmosphere with 5% CO_2_, cells were harvested, and viability was assessed using the trypan blue exclusion method with a TC-20 cell counter (Bio-Rad, Hercules, CA, USA). Cultures consistently maintained a viability rate of 90% or higher. For selected experiments, RAW cells were cultured under basal conditions without additional stimuli. Cells collected immediately after seeding were defined as time zero (T0). Parallel cultures were maintained and harvested at 24-, 48-, and 72-hour post-seeding. These time points were utilized as unstimulated control conditions for subsequent analyses.

### 2.3 Flow cytometry

The expression of CD3, CD4, CD8, CD28, and TCRβ was assessed using multiparametric flow cytometry in both unstimulated RAW cells and cells stimulated with anti-CD3/IgG for 5 and 24 hours. In an additional model, stimulation was analyzed over a 24–72-hour time frame to monitor changes in the expression of key markers. Cells were harvested and stained at 4 °C for 20 minutes with fluorochrome-conjugated monoclonal antibodies (mAbs) targeting CD3, CD4, CD8, CD28, TCRβ, and F4/80 (BioLegend, San Diego, CA, USA). After staining, fixation procedures were applied prior to flow cytometry analysis. Cell acquisition was performed using a FACS Aria II flow cytometer (Becton Dickinson, Franklin Lakes, NJ, USA) with single-fluorochrome samples for compensation. Data were analyzed with FlowJo software (Tree Star, San Carlos, CA, USA). Fluorescence-minus-one (FMO) controls were used alongside isotype controls to determine background staining levels. Dead cells were excluded based on side scatter/forward scatter gating, and approximately 50,000 events were recorded per sample.

### 2.4 RAW cells stimulation *in vitro*

Designated wells were pre-coated with anti-CD3 antibody (1 µg/mL) and IgG2a to stimulate Fc receptors or with isotype controls (BioLegend, San Diego, CA, USA) in sterile PBS. Plates were incubated overnight at 37 °C in a humidified atmosphere with 5% CO_2_. On the day of the experiment, unbound antibodies were removed. A total of 5 × 10^5^ RAW cells, either unsorted or sorted, were plated and cultured for 24 hours in RPMI 1640 medium (GIBCO, Grand Island, NY, USA) supplemented with 2 mM L-glutamine, 100 µg/mL streptomycin, 100 IU/mL penicillin, and 10% FBS (GIBCO, Grand Island, NY, USA). The cells were maintained at 37 °C with 5% CO_2_. After 24 hours, supernatants and cell pellets were collected for downstream analyses, including enzyme-linked immunosorbent assays (ELISA) and Western blotting (WB).

To induce nutrient stress, RAW cells were cultured under conditions of reduced serum availability. Cells were maintained in RPMI 1640 medium supplemented with fetal bovine serum (FBS) at final concentrations of 10% (standard condition), 1%, or 0.1%. In parallel, an additional nutrient-stress condition was established by culturing cells in RPMI 1640 supplemented with 10% FBS but lacking sodium pyruvate. Unless otherwise indicated, cells were seeded at identical densities and maintained under these conditions for the specified time points (T0, 24, 48, and 72 hours). These experimental settings were employed to assess the impact of nutrient deprivation on CD3 expression and subsequent signaling responses.

### 2.5 Phagocytosis assay

Phagocytic activity was quantified utilizing the Phagocytosis Assay Kit (IgG-FITC) (Cayman Chemical, Ann Arbor, MI, USA), following the manufacturer’s guidelines. A total of 5 × 10^5^ RAW cells were seeded and cultured for 4 hours in supplemented RPMI 1640 medium. At the conclusion of the cell culture, cells were incubated with a 1:200 dilution of latex bead–rabbit IgG–FITC complexes at 37 °C for 1 hour. Subsequently, cells were washed with PBS, detached with 1% trypsin-EDTA (Invitrogen, Carlsbad, CA, USA), and the reaction was halted by adding DMEM. Cell suspensions were transferred to microtubes, centrifuged at 8000 × g for 8 minutes at 4 °C, and resuspended in PBS with 1% Paraformaldehyde. These cells were stained at 4 °C for 20 minutes with fluorochrome-conjugated mAbs targeting CD3 and F4/80 (BioLegend, San Diego, CA, USA). Single-cell fluorescence was quantified by flow cytometry (10,000 events per sample) and compared between treated cells and control cells lacking beads (non-phagocytic control). Data acquisition was performed using a flow cytometer (FACSAria II, BD Biosciences), and the results were normalized by setting the mean percentage of positive cells in the control condition to 100%.

### 2.6 Splenocytes from C57BL/6 mice

Wild-type male C57BL/6 mice (8–12 weeks old) were housed in the animal facility of the Instituto. All animal procedures were conducted in accordance with institutional guidelines and received approval from the Institutional Committee for the Care and Use of Laboratory Animals. Splenocytes were isolated from mechanically dissociated spleen tissue and utilized as a positive control for CD3 expression.

### 2.7 RNA extraction and qPCR

RAW cells were collected at 0, 24, 48, and 72 hours after anti-CD3/IgG2a stimulation. The cells were suspended in DNA/RNA Shield solution (Zymo Research, Irvine, CA, USA) and stored at -70 °C until RNA extraction. Total RNA was isolated using the RNeasy Micro Kit (Qiagen, Hilden, Germany), with genomic DNA eliminated using the RNA-Free DNase Set (Qiagen, Hilden, Germany), in accordance with the manufacturer’s protocols. Nucleic acid quantification was performed utilizing the Qubit™ assay kit and a Qubit 2.0 Fluorometer (Life Technologies, Waltham, MA, USA). RNA extracted from splenocytes was obtained following the same technical procedures.

For cDNA synthesis, 300 ng of RNA from RAW cells or splenocytes was converted into cDNA using the High-Capacity cDNA Reverse Transcription Kit (Applied Biosystems, Waltham, MA, USA) in a 20 µL reaction, according to the manufacturer’s instructions.

Relative gene expression was evaluated through quantitative real-time PCR (qPCR) utilizing TaqMan probes or SYBR Green dye. The following probes were employed for murine genes: *CD3* (CD3e gene) (Mm01179194_m1), *IRF1* (Interferon Regulatory Factor 1) (Mm01288580_m1); *Mafb* (v-maf musculoaponeurotic fibrosarcoma oncogene family-protein B) (Mm00627481_s1), *GATA3* (GATA binding protein 3) (Mm00484683_m1), *CCL5* (Chemokine (C-C motif) ligand 5) (Mm01302427_m1), *18S* (Rn18s, Mm03928990_g1), and *ACTB* (β-actin) (Mm00607939_s1). The specific forward and reverse primers, as documented in the literature (11), were utilized for the amplification of *HIF1a* (Hypoxia-Inducible Factor 1-alpha), namely 5′-CCTGCACTGAATCAAGAGGTTGC-3′ and 5′-CCATCAGAAGGACTTGCTGGCT-3′; and for *ACTB*, 5′-CAGGTCATCACTATTGGCAA-3′ and 5′-AGGTCTTTACGGATGTCAAC-3′.

Individual reactions were prepared using 7.5 ng of cDNA template in conjunction with either the Maxima Probe/ROX qPCR Master Mix or the SYBR Green PCR Master Mix (Thermo Fisher Scientific, Waltham, MA, USA). The amplification procedures were carried out in duplicate following the subsequent thermal cycling parameters: an initial denaturation at 95 °C for 10 minutes, followed by 40 cycles consisting of 60 °C for 1 minute and 95 °C for 15 seconds, performed in a Step One Plus Real-Time PCR System (Applied Biosystems, Carlsbad, CA, USA). A final melting curve analysis was conducted to confirm the specificity of the amplified products via SYBR Green qPCR.

Relative gene expression (RE) was determined using the ΔΔCT method to calculate the fold change for each target gene under different experimental conditions. Results were normalized to endogenous controls (*ACTB* and *18S*). Gene expression was calculated relative to the reference group: unstimulated RAW cells for anti-CD3 effects at different time points, uninfected RAW cells for BCG infection, or a standard condition for nutrient stress. The 2^−^ΔΔCT = 1 method was used as the baseline. For comparisons of *CD3*, *IRF1*, *Mafb*, and *GATA3* gene expression, ΔCT values were normalized to species-specific housekeeping genes, and relative expression levels were calculated using uninfected MDM as the reference.

### 2.8 Western blot

The cells were lysed on ice for 10 minutes in Pierce RIPA buffer (Thermo Scientific, Waltham, MA, USA), supplemented with phosphatase and protease inhibitors (Roche, Basel, Switzerland). Protein concentrations in the lysates were subsequently quantified using the Pierce BCA Protein Assay Kit (Thermo Scientific, Waltham, MA, USA) in accordance with the manufacturer’s instructions. The lysates were then denatured by boiling for ten minutes in 4× SDS Laemmli sample buffer.

Subsequently, 20–25 µg of protein extract was separated by SDS-PAGE, transferred onto nitrocellulose membranes, and subjected to immunoblotting with rabbit monoclonal antibodies (mAbs) specific to NFAT (NFAT1, D43B1 Cat. No. 5861, Cell Signaling), IKK-β (IKK beta, J.414.5- MA5- Cat. No. 15080, InvitroGen), pC-Jun (Phospho-c-Jun, Ser73, D47G9, Cat. No. 3270, Cell Signaling), NF-κB (NF-kappaB p65, D14E12, Cat. No. 8242, Cell Signaling), and STAT-1 (STAT1-79, Cat. No. AHO0832, InvitroGen). Housekeeping proteins, including β-actin (beta-actin, Cat. No. GTX109639, GeneTex), tubulin (Tubulin, YL1/2, Cat. No. GTX76511, GeneTex), or GAPDH (GAPDH, ZG003, Cat. No. 39-8600, InvitroGen), were used as loading controls to ensure equal protein loading.

Western blot data were analyzed using Image Lab software V.6.1 (Bio-Rad, Hercules, CA, USA). Protein bands were defined using volume boxes for both the sample and control groups. Data were normalized to housekeeping protein levels for relative comparisons across different culture conditions.

### 2.9 Cytokine measurement in cell culture supernatants

Cell culture supernatants were thawed and analyzed using a bead-based multiplex assay to quantify cytokines (BioLegend, San Diego, CA, USA). This panel allows simultaneous quantification of 8 mouse cytokines, including IL-5, IL-13, IL-2, IL-6, IL-10, IFN-γ, TNF-α, and IL-4 (Legendplex Cat. No. 741053). The assay was performed according to the manufacturer’s protocols, and samples were analyzed using a BD Accuri™ C6 Plus Flow Cytometer (Becton, Dickinson and Company, Franklin Lakes, NJ, USA). Acquired data were analyzed using the online data analysis software suite from *Qognit* (BioLegend, San Diego, CA, USA).

### 2.10 Statistical analysis

Statistical analyses were conducted utilizing GraphPad Prism version 11 (GraphPad Software, La Jolla, CA, USA). The results are expressed as mean ± standard deviation (SD), unless specified otherwise. For datasets not conforming to a normal distribution, multiple-group comparisons were executed using the Kruskal–Wallis test, followed by Dunn’s *post hoc* test for multiple comparisons.

When data represented the mean of independent experiments, results were expressed as mean ± standard error of the mean (SEM). In these cases, statistical comparisons among multiple groups were performed using one-way analysis of variance (ANOVA) followed by Bonferroni’s *post hoc* test. The number of independent experiments (n) is indicated in the corresponding figure legends. A p-value < 0.05 was considered statistically significant.

## 3 Results

### 3.1 RAW cells express T-cell-associated receptors, and the CD3 expression is influenced by time and nutrient deficiency

**Figure 1:**
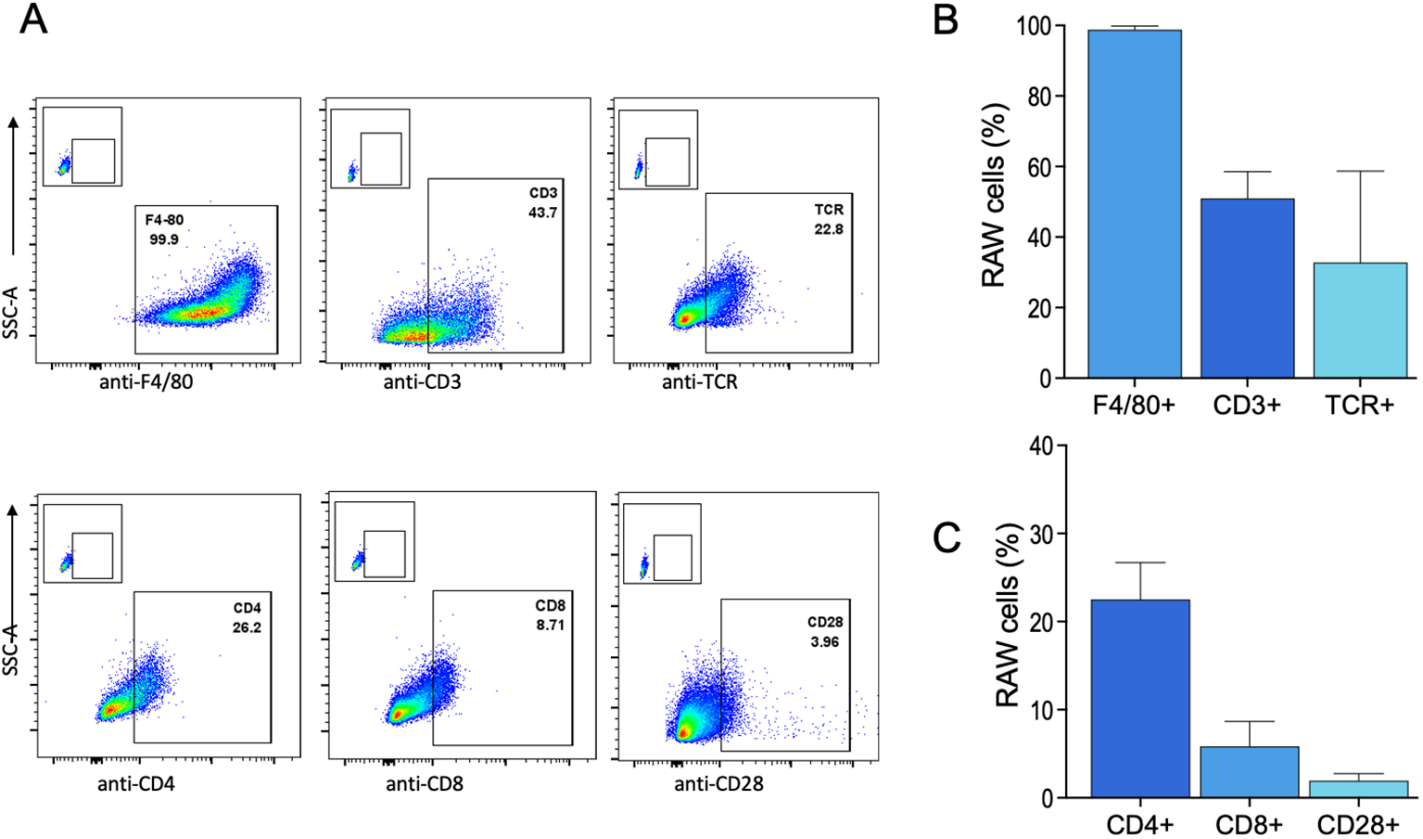
Expression of TCR-associated molecules in RAW cells. RAW cells were cultured for 24 hours and prepared for flow cytometric analysis. Representative dot plot of the gating strategy; the small image on the upper-left side is the negative fluorescence control **(A)**. The frequency of RAW cells expressing F4/80, CD3, and TCR **(B)**, and CD4, CD8, and CD28 **(C)**. Bars represent the mean percentage of positive cells ± SD from ten independent experiments.

Previous data indicated that RAW cells express T-cell function-related receptors (9, 10). First, using flow cytometry, we confirmed that T-cell receptors are expressed on RAW cells (**Figure 1A**). Our findings showed that 51% of RAW cells are CD3+, and a lower frequency are TCR+ (32%) (**Figure 1B**). When other T-cell receptors were evaluated, we identified that 22% of RAW cells are CD4+, whereas the frequency of CD8+ and CD28+ is very low (2 and 5%, respectively) (**Figure 1C**).

**Figure 2:**
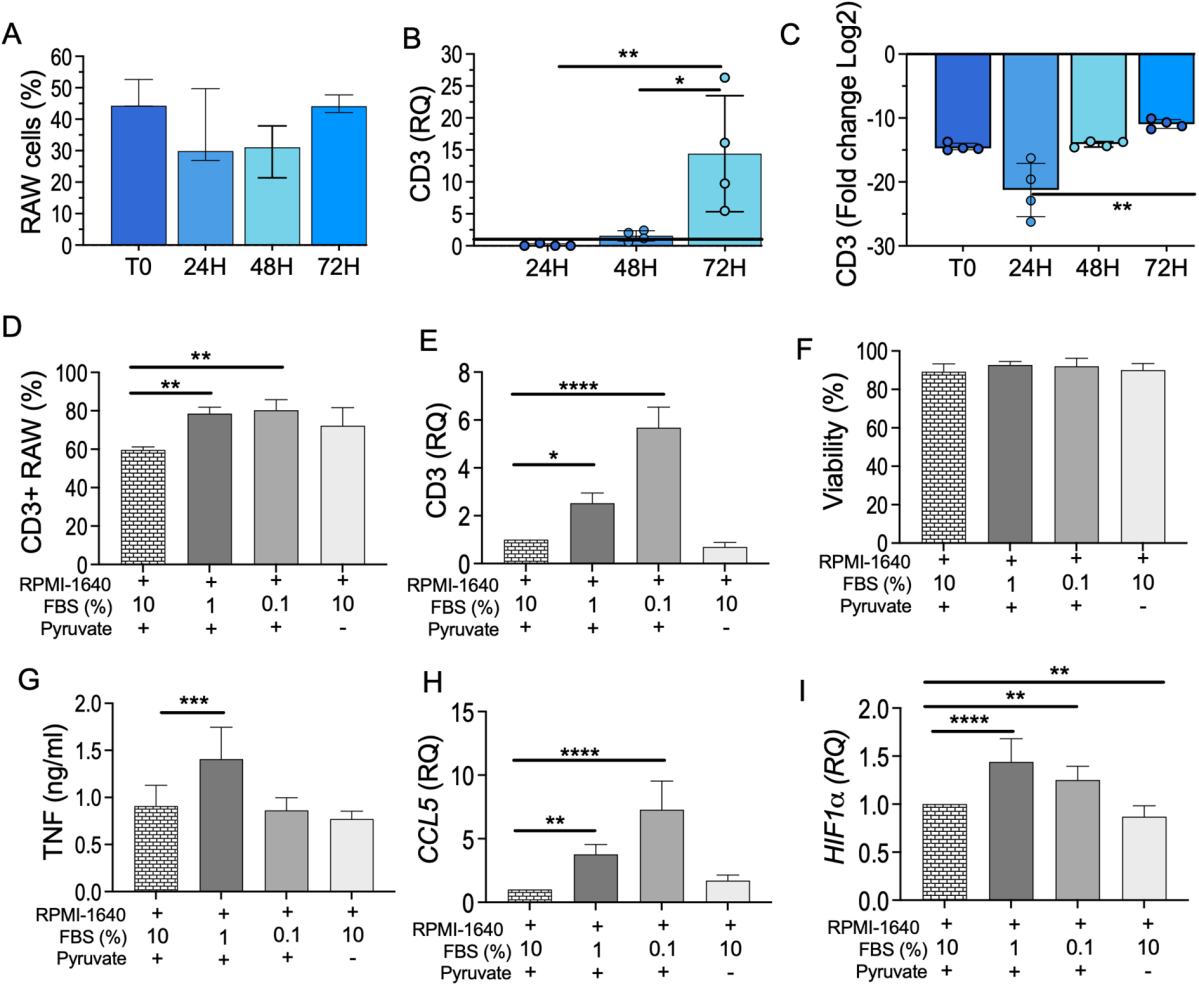
The CD3 expression on RAW cells is modulated by time and nutrient deficiency. Flow Cytometry evaluated RAW cells for up to 72 hours for CD3 **(A)**. Relative expression of CD3 was assessed by qPCR for up to 72 hours **(B)**, and the relative fold change across time is shown **(C)**. Other cultures were maintained under FBS restriction or pyruvate deprivation for 24 hours to assess changes in CD3 expression at both the protein level by flow cytometry **(D)** and the transcriptional level by qPCR **(E)**. The live cell percentage of these cultures was determined by flow cytometry **(F)**. Additionally, TNF levels were quantified in the culture supernatant **(G)**, and *CCL5* and *HIF1α* transcriptional levels were evaluated by qPCR **(H, I)**, respectively). Data shown were represented as mean ± SD (A-C, n=4; D-G, n=8); asterisks indicate the statistically significant difference between the compared conditions: ∗p < 0.05; **p < 0.01; ***p<0.001; ****p<0.0001.

Subsequently, we assessed whether temporal factors influence the modulation of CD3 expression. Flow cytometry analysis demonstrated a marginal decrease in CD3 protein levels at 24- and 48-hour post-culture, though these changes were not statistically significant (**Figure 2A**). Conversely, at the transcriptional level, CD3 expression exhibited temporal variation; T0 was established as the baseline, normalized to 1 (black line), at 24 hours, there was a slight reduction (mean 0.1), while at 72 hours post-culture, CD3 expression increased markedly, with significant differences compared to both 24 and 48 hours (means: 13 vs. 0.1, p<0.01 and 1.6, p<0.05; respectively) (**Figure 2B**).

Subsequently, we assessed whether the elevated CD3 expression in RAW cells matched that typically expressed by T lymphocytes. The transcriptional CD3 expression in RAW cells was compared with that in murine splenocytes, with the latter set to zero. Our data demonstrated that after 24 hours, CD3 expression decreased 21-fold, but after 72 hours, it decreased 10-fold, indicating that, following 72 hours of culture, RAW cells increased their CD3 expression compared to 24-hour levels (p<0.01). Furthermore, the expression level approached that observed in splenocytes (**Figure 2C**).

To assess whether the timing influences CD3 expression at the transcriptional level in relation to nutrient availability, RAW cells were cultured for 24 hours under conditions of FBS or pyruvate deficiency, which induce significant global cellular stress. Our data confirmed that, relative to the standard condition (10% FBS), CD3 expression at the protein level increased under conditions of 1% or 0.1% FBS (means: 79%, p<0.01; 80%, p<0.01, respectively) (**Figure 2D**). Similar findings were observed at the transcriptional level, where the standard SFB condition was normalized to 1, and both 1% and 0.1% FBS elevated CD3 expression (means: 2.5, p<0.05; 6.8, p<0.0001, respectively) (**Figure 2E**). It is noteworthy that our data are not overstated, as cell viability remained consistently above 90% across all conditions (**Figure 2F**).

Finally, we confirmed that despite the stress condition, these cells maintain their capacity to produce pro-inflammatory molecules. Our findings indicated that, in comparison with the standard condition, 1% FBS increased TNF levels in the culture supernatant (**Figure 2G**). At the transcriptional level, the standard condition (10% FBS) was normalized to 1; *CCL5* expression increased under conditions of 1 and 0.1% FBS (means: 3.7, p<0.01; 7.2, p<0.0001, respectively) (**Figure 2H**); whereas both FBS and pyruvate deprivation influenced HIF1α transcriptional levels. Under 1% and 0.1% FBS, levels increased to 1.4 and 1.2, respectively (p<0.0001 and p<0.01, respectively). Conversely, pyruvate deprivation decreased *HIF1α* levels to 0.85 (p<0.01) (**Figure 2I**). Collectively, our findings suggest that under these nutrient-deprivation conditions, CD3 is upregulated, thereby maintaining viable CD3+ RAW cells capable of secreting a pro-inflammatory profile.

### 3.2 CD3+ RAW cells exhibit efficient phagocytic capacity

**Figure 3:**
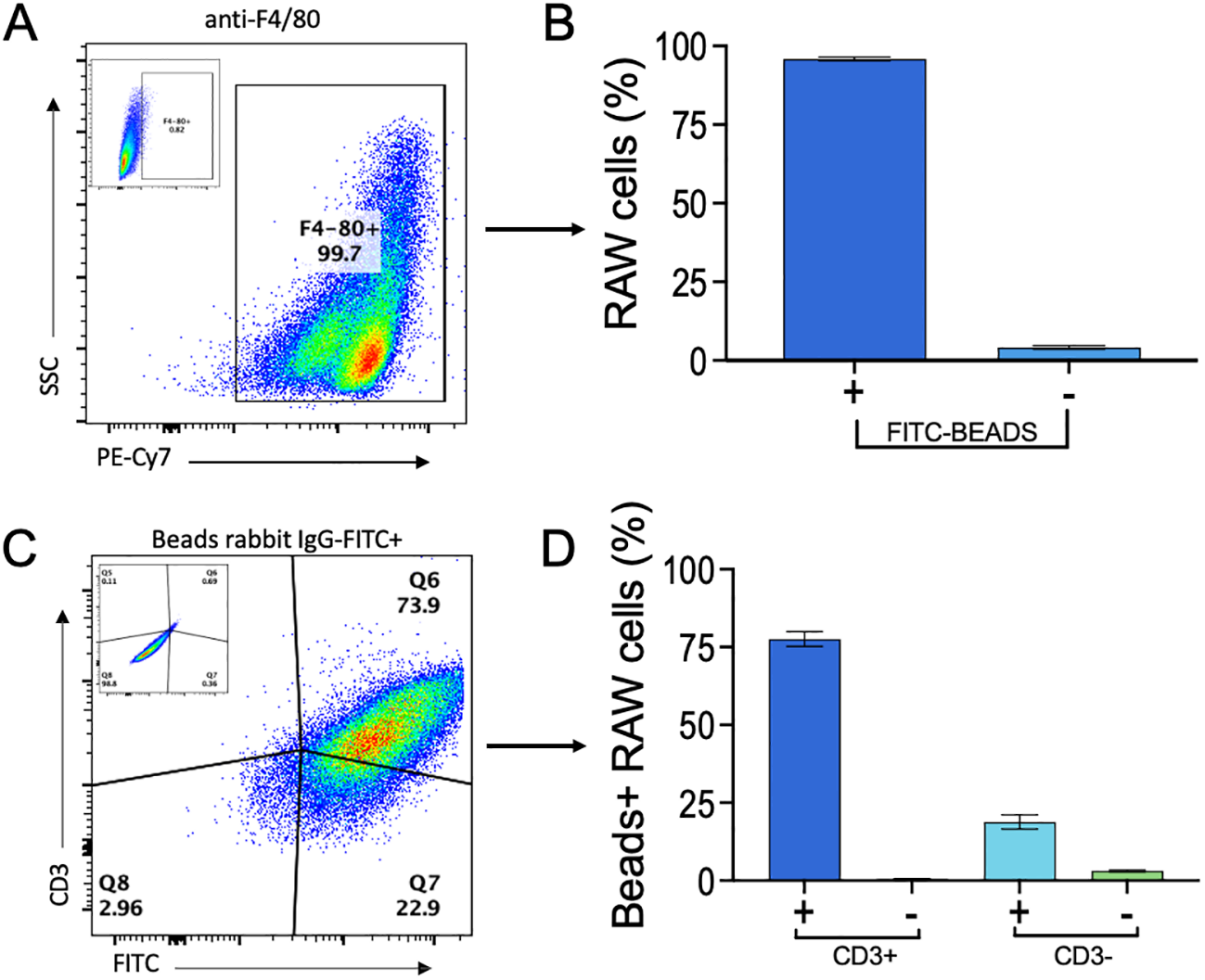
CD3+ RAW cells efficiently phagocyte synthetic beads. RAW Cells evaluated by Flow Cytometry, cells were cultured for up to 1 hour with FITC-BEADS for phagocytosis assay. Representative dot plots of the gating strategy; the small image on the upper-left side is the negative fluorescence control of analysis for F4/80 **(A)** and IgG-FITC+ latex beads **(C)**. The frequency of RAW that makes phagocytosis is shown in **(B)**. The frequencies of CD3+ and CD3- cells that phagocytose and the fraction that do not phagocytose are compared, and the data are shown in **(D)**. Bars represent the mean percentage of positive cells ± SD from eight independent experiments.

Phagocytosis is one of the primary functions of macrophages; thus, we evaluated the phagocytic capacity of CD3+ RAW cells. A latex bead-based assay was conducted and analyzed by flow cytometry. First, F4/80+ cells were delimited (**Figure 3A**), and then, the frequency of RAW cells that phagocytosed latex beads (FITC+) was determined. The results showed that 96% of RAW cells were positive to beads (**Figure 3B**). Subsequently, we evaluated which RAW cell subsets (CD3+ or CD3-) displayed a better phagocytosis ability (**Figure 3C**). Data indicated that 78% of RAW cells were CD3+ beads+ and approximately 2% were CD3+ beads-, whereas around 20% are CD3- beads+ and 3% of CD3- beads- cells, suggesting that only a minimal RAW cell did not exhibit phagocytic activity (**Figure 3D**).

These findings suggest that CD3+ RAW cells exhibit an optimal capacity for phagocytosis, comparable to their CD3- counterparts. This highlights that, regardless of whether these phagocytic cells are classified as classical (CD3-) or non-classical (CD3+), they maintain optimal functionality as subpopulations within innate immune responses.

### 3.3 RAW cells deliver a proinflammatory profile mediated by CD3 and FcγR signaling

Following the assessment of the functional capacity of CD3+ RAW cells, and considering the recent suggestion that FcγR-II/III receptors provide a co-stimulatory signal in CD3+ RAW cells (10), the cytokine profile induced by the CD3/FcγR-II/III-dependent pathway was examined at 5, 24, 48, and 72 hours.

**Figure 4:**
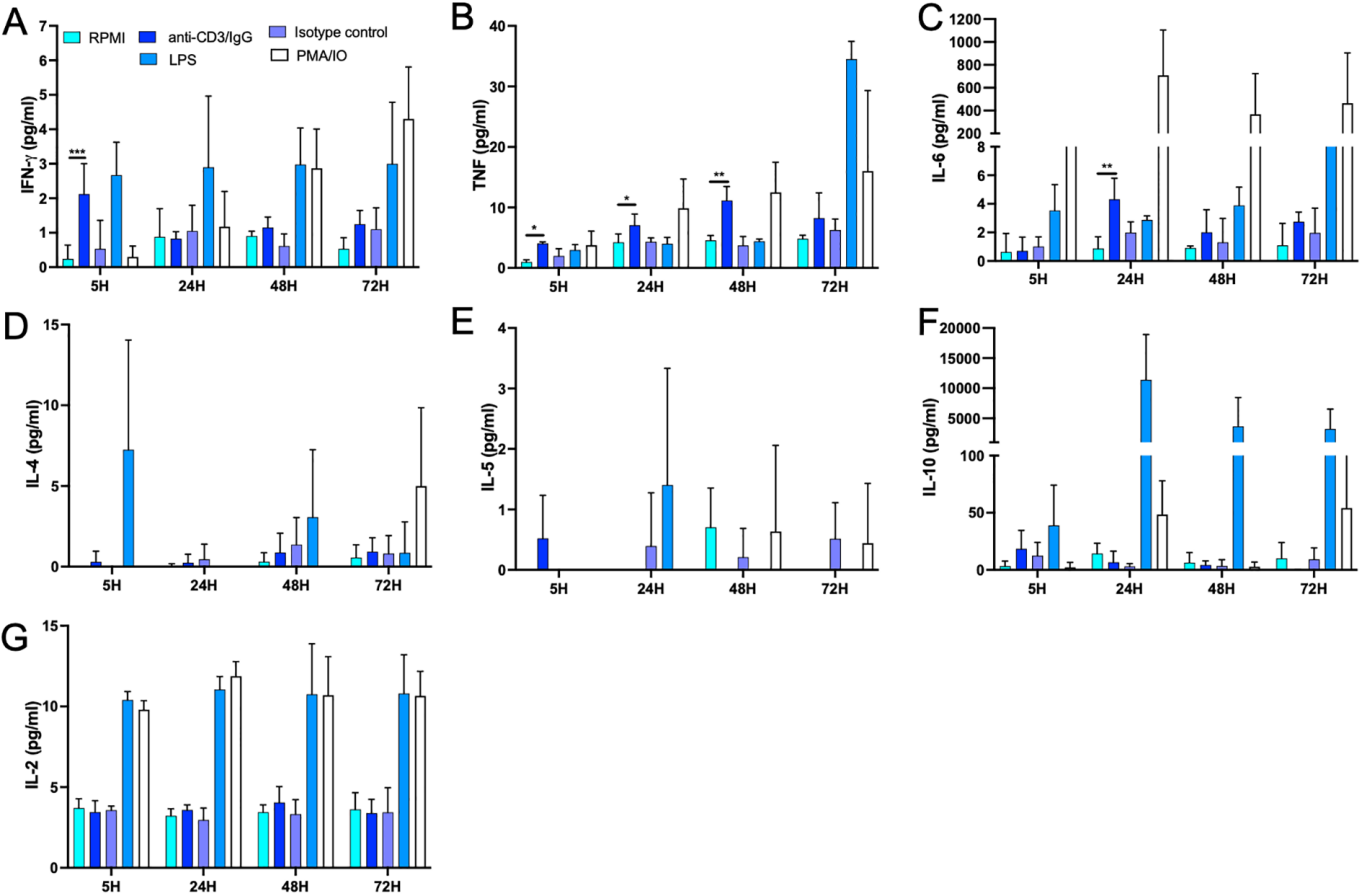
Cytokine production of RAW cells after stimulation of the CD3 complex and Fc-γR. Cytokine levels were evaluated using a bead-based multiplex assay in the supernatants obtained from cell cultures with different experimental conditions: Unstimulated as control conditions (RPMI), stimulated with anti-CD3 + IgG2a (anti-CD3/IgG), isotype antibody controls (Isotype control), and a positive known stimulus such as Lipopolysaccharide (LPS) or PMA-Ionomycin (PMA/IO). Levels of the proinflammatory IFN-γ **(A)**, TNF **(B)**, and IL-6 **(C)**; the antiinflammatory IL-4 **(D)**, IL-5 **(E)**, and IL-10 **(F)**; and IL-2 **(G)**. Data were shown as mean ± SD (n=8); asterisks indicate the statistically significant differences between the compared conditions: *p < 0.05; ** p < 0.01; *** p < 0.001.

Our findings demonstrate that, in comparison with unstimulated conditions, CD3-dependent stimulation favored a predominantly proinflammatory profile, with IFN-γ production observed as early as 5 hours (p<0.001) (**Figure 4A**). Furthermore, TNF production showed a significant increase from 5 to 48 hours (5 hours: p<0.05; 24 hours: p<0.05; 48 hours: p<0.01) (**Figure 4B**), while IL-6 levels reached a notable peak at 24 hours (p<0.01) (**Figure 4C**). The anti-inflammatory profile, consisting of IL-4, IL-5, and IL-10 levels, was not elicited via the CD3-dependent pathway (**Figures 4D–F**); similarly, IL-2 was not induced (**Figure 4G**). For clarity, statistical differences attributable to positive stimuli (LPS and PMA/IO), which are expected to induce a clear proinflammatory profile, were not reported to emphasize the cytokine profile solely induced by the CD3-dependent pathway.

These findings collectively reinforce the concept that RAW cells effectively promote a proinflammatory profile, facilitated through CD3 and FcγR signaling pathways.

### 3.4 CD3 and FcγR signaling activate the early NFAT pathway and, later, NF-κB in RAW cells

**Figure 5:**
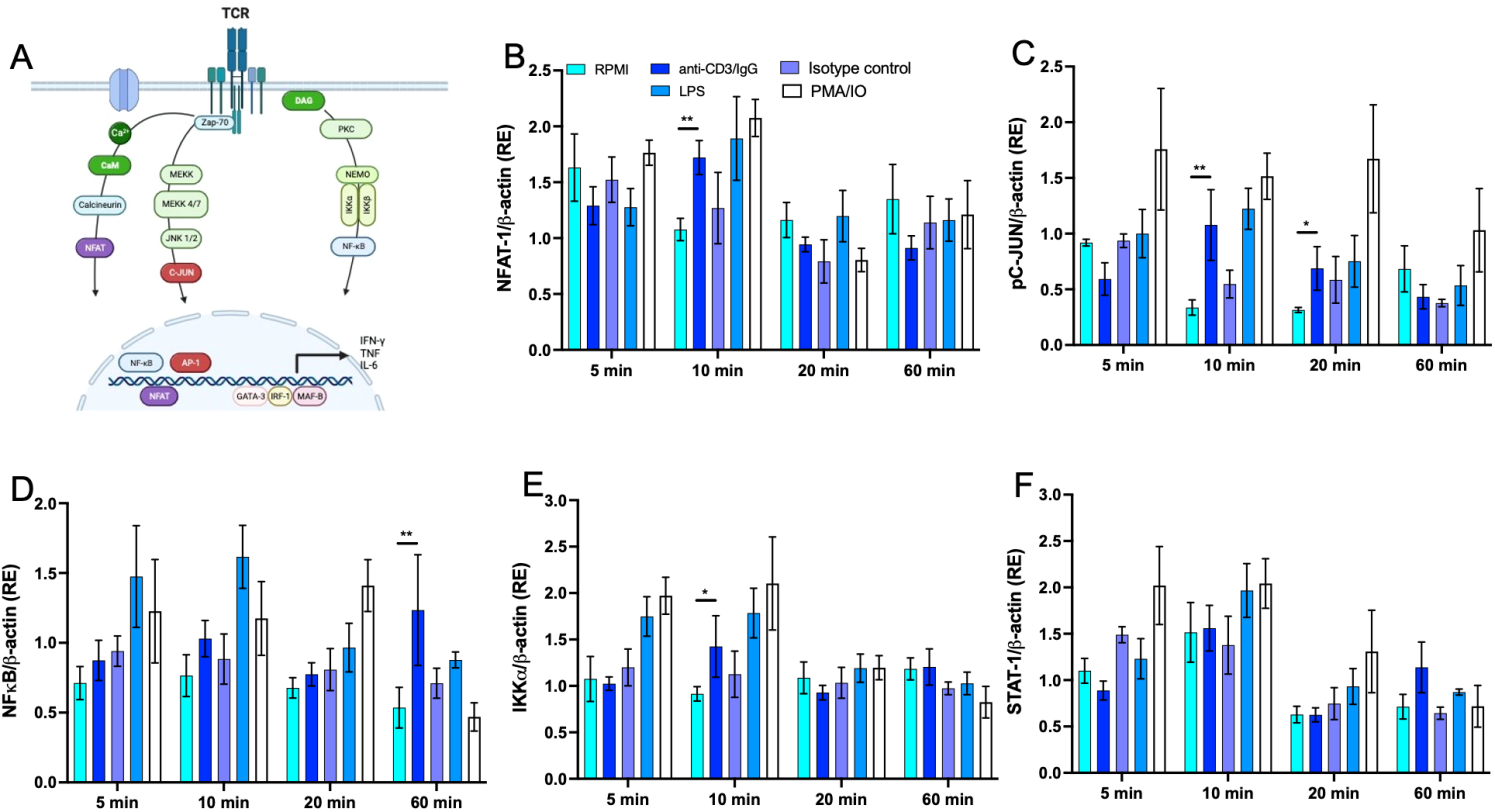
Anti-CD3/IgG2a activates signaling pathways that involve NF-kB, NFAT-1, and C-Jun. A simplified schematic representation of signaling pathways activated downstream of the TCR–CD3 complex is shown in **(A)**, with the figure created at https://BioRender.com. RAW cells were cultured under different experimental conditions, and whole-cell lysates were analyzed by Western blot to evaluate the relative expression (RE) of NFAT-1 **(B)**, p-c-Jun **(C)**, NF-κB **(D)**, IKK-α **(E)**, and STAT-1 **(F)**. Cells were cultured under unstimulated conditions (RPMI) as a basal control, stimulated with anti-CD3 plus IgG2a (anti-CD3/IgG), treated with isotype control antibodies (isotype control), or stimulated with positive controls such as lipopolysaccharide (LPS) or PMA plus ionomycin (PMA/IO). β-Actin was used as a loading control. Data are presented as mean ± SD from six independent experiments (n = 6). Statistical significance between experimental conditions is indicated as follows: *p < 0.05; **p < 0.01.

Considering molecules classically associated with CD3-dependent signaling in T cells (**Figure 5A**), we analyzed molecules induced in RAW cells by co-stimulation of CD3 and Fc-γR using Western blotting (see **Supplementary Figure 1** for a representative blot). Signaling and phosphorylation events typically occur within a range from a few minutes to the early hours following stimulation. Then, STAT-1, IKK-α, NFAT-1, NF-κB, and pC-Jun were evaluated at 5-, 10-, 20-, and 60-minute post-stimuli. Longer times (5 and 24 hours) were also evaluated, but as expected, no significant changes were observed (**Supplementary Figure 2**).

Our results revealed that, in comparison with unstimulated conditions, anti-CD3 + IgG increased NFAT-1 after 10 minutes post-stimulus (p<0.01) (**Figure 5B**). Although pC-Jun levels also increased after 10 minutes, they were maintained at a higher level until 20 minutes (p<0.01 and p<0.05, respectively) (**Figure 5C**). Concerning the NFκB pathway, the anti-CD3 + IgG stimulus initially increased IKKα/β at 10 minutes and later, at 60 minutes, NFκB levels increased (**Figures 5D, E**). The level of STAT-1 remained unchanged (**Figure 5F**). As with the previous figure, statistical differences attributable to positive stimuli (LPS and PMA/IO) were not reported to highlight the cytokine profile exclusively induced by the CD3-dependent pathway.

These findings suggest that within the first 10 minutes after a stimulus, the CD3-dependent pathway initiates signaling involving NFAT-1 and C-Jun; however, NF-κB activation occurs later.

### 3.5 CD3 and FcγR signaling activate the IRF-1 transcription factor and suppress MAFB in RAW cells

Finally, we evaluated transcription factors that promote the functional inflammatory profile in macrophages by a CD3-dependent pathway.

**Figure 6:**
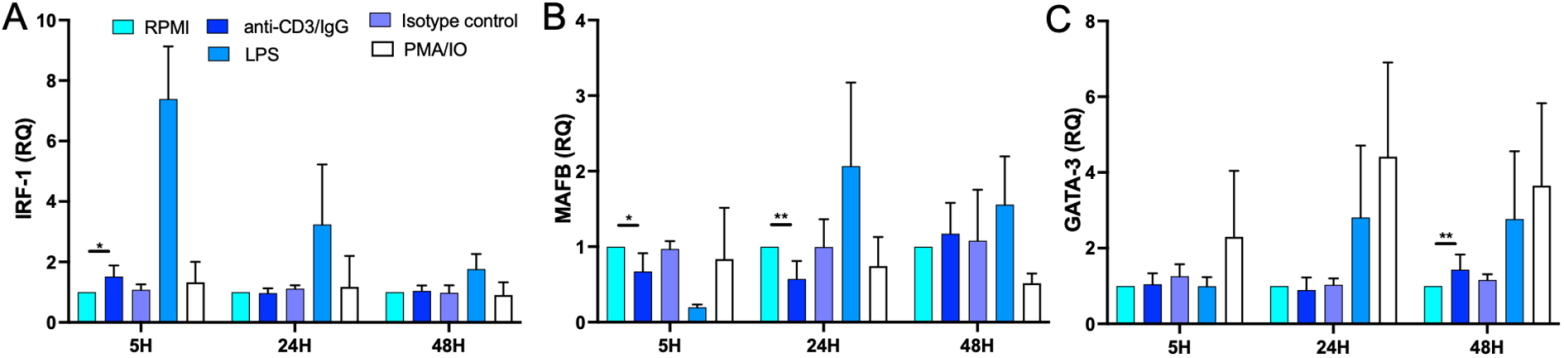
Anti-CD3/IgG2a stimulation modulates the expression of transcription factors in RAW cells. RAW cells were cultured under different experimental conditions, and the relative gene expression of transcription factors was quantified by qPCR at 5, 24, and 48 h. The relative quantification (RQ) of the *IRF-1* **(A)**, *MAFB* **(B)**, and *GATA-3* gene expression **(C)** was determined in cells maintained under unstimulated conditions (RPMI) as basal control, stimulated with anti-CD3 plus IgG2a (anti-CD3/IgG), treated with isotype control antibodies (isotype control), or stimulated with positive controls such as lipopolysaccharide (LPS) or PMA plus ionomycin (PMA/IO). Data are presented as mean ± SD from five independent experiments (n = 5). Statistical significance between conditions is indicated as follows: *p < 0.05; **p < 0.01.

Our findings indicate that, relative to unstimulated conditions, the anti-CD3 + IgG2a stimulus elevated *IRF-1* levels, a proinflammatory transcription factor, at 5 hours post-stimulation (p<0.05). By 24 and 48 hours, *IRF-1* levels had returned to baseline (**Figure 6A**). Consistently, the levels of *MAFB*, an anti-inflammatory transcription factor, remained lower than those in unstimulated samples at both 5 and 24 hours (p<0.05 and p<0.01, respectively), and normalized at 48 hours (**Figure 6B**). Conversely, *GATA-3* levels did not exhibit early downregulation; instead, they increased at later time points, with higher levels observed at 48 hours post-stimulation compared to unstimulated controls (p<0.01) (**Figure 6C**). As with previous figures, the positive stimuli elicited activation of transcription factors; however, the statistical significance of these differences is not emphasized to highlight the specific profile induced by the CD3-dependent pathway.

Together, these findings demonstrated that within a short timeframe, the CD3 stimulus elicited a proinflammatory profile in RAW cells, mediated by increased *IRF-1* and suppression of *MAFB*.

## 4 Discussion

The RAW 264.7 cell line is extensively employed as a model in biomedical research to investigate the mechanisms underlying innate immune responses. These cells demonstrate significant plasticity and can rapidly undergo polarization and differentiation in response to stimuli such as lipopolysaccharide (LPS), thereby facilitating assessment of critical cellular processes, including phagocytosis, the production of inflammatory mediators, and interactions with other immune system components (12). An intriguing and less well-characterized phenotype of RAW cells is their ability to express CD3 and respond to polyclonal stimulation, resulting in the expression of anti-CD3 and Fc receptors (9, 10). In this study, we aim to explore the functional expression and signaling capabilities of the CD3 complex in RAW cells, thereby supporting the hypothesis that these cells may utilize aspects of T cell signaling machinery.

Following the examination of CD3 and TCR on RAW cells, consistent with previous studies indicating a hybrid immunophenotype within this myeloid population (10), we observed that CD4, a membrane glycoprotein, was predominantly expressed on the surface of RAW cells. Consistent with previous reports, we found a hybrid immunophenotype in RAW cells, with expression of CD3 and TCR and low levels of CD4. Although CD4 is classically associated with T cells, low-level CD4 expression has also been reported in subsets of human monocytes and macrophages under inflammatory conditions, and it has been proposed that CD4 expression in this monocyte subset may induce cytokine production and facilitate differentiation into functionally mature macrophages (13). The presence of CD4 in RAW cells further supports the notion that these cells exhibit features that overlap innate and adaptive immune phenotypes.

The functional significance of CD4 expression was not directly addressed in the present study. Therefore, our findings should be interpreted as evidence of phenotypic plasticity in RAW cells rather than proof of a specific CD4-dependent signaling mechanism. Future studies will be required to determine whether CD4 contributes to the activation or differentiation programs of this macrophage population. Conversely, the expression of either CD8 or CD28 was less than 5%, thereby confirming that this co-stimulatory molecule is expressed at negligible levels and reinforcing the hypothesis that the CD3-dependent signaling pathway in myeloid cells differs from the classical pathway observed in T cells.

CD3 expression exhibits considerable variability, functioning as a dynamic marker that changes in response to both the duration of cell culture and, more notably, the activation status of T cells (14). In our model, CD3 expression in RAW cells was normalized at the transcriptional level after 72 hours of culture, similar to the dynamic changes in CD3 observed in T cells. This comparison supports the presence of a partially T cell-like phenotype in RAW cells and highlights a high degree of plasticity in the marker expression that influences cell activation. Our findings reinforce the value of RAW cells as a model for studying noncanonical CD3/TCR signaling in macrophages (9) and suggest further exploration of the functional relevance of these receptors within innate immune responses.

It is well established that under conditions of hypoxia and nutrient deprivation, T cells can upregulate surface markers such as PD-1, CTLA-4, and A2aR, while showing decreased IFN-γ expression (15). Notably, deprivation of L-Arg (also referred to as “starvation”) during extended culture periods functions as a nutritional stressor, rapidly inducing downregulation of the T cell antigen receptor ζ chain (CD3ζ), along with concurrent alterations in protein and mRNA levels in Jurkat cells (16). Starvation induced by fetal bovine serum (FBS) primarily affects transcription factors through degradation mediated by starvation-induced autophagy, thereby reducing cellular protein levels (17). Nevertheless, the specific effect of starvation on CD3 expression in RAW cells remains unclear and warrants further investigation. To explore this aspect, we investigated whether conditions of metabolic stress could modulate CD3 expression. Our findings indicate that restriction of serum and pyruvate substantially elevates both CD3 mRNA and surface protein levels, suggesting that nutrient deprivation may serve as an environmental cue that promotes CD3 upregulation in RAW cells.

These findings align with prior evidence indicating that macrophage phenotypes are highly responsive to external stressors, thereby further substantiating the hypothesis that CD3 may play a role in cellular responses to metabolic challenges (18). In the THP-1 model, the FXR1 protein has been identified as a key regulator promoting the expression of specific cytokines and chemokines, thereby facilitating cell migration (19). Our results demonstrate that RAW cells exhibit a significant increase in TNF levels following FBS deprivation; this proinflammatory profile is consistent with previously reported data (6). Furthermore, we confirmed that FBS deprivation induces classical stress-associated markers, including regulators of cytokines and chemokines, as observed in the THP-1 model. In our model, *HIF-1α* levels were elevated; this molecule is regulated by oxygen availability and nutrient status. mTORC1 promotes *HIF-1α* translation; however, during nutrient starvation, mTORC1 activity is inhibited, thereby activating stress pathways such as GCN2–ATF4, which leads to increased transcription of *HIF-1α* (20). Similarly, inhibition of mTORC1 and cellular stress induced by nutrient deprivation activate GCN2, ATF4, and NF-κB, resulting in the expression of proinflammatory chemokines (21). Our study further indicates that nutrient deprivation promotes *CCL5* expression.

We support the hypothesis that nutrient deprivation induces, at least in the RAW cell model, an increase in CD3, with these cells maintaining a proinflammatory functional profile; however, it is well known that the metabolic consequences are complex and multifaceted. Nutrient limitation can activate several stress−responsive pathways, including autophagy, mTOR inhibition, AMPK activation, and oxidative stress programs, each of which can independently modulate inflammatory signaling. This study did not clarify if the increased CD3 expression reflects a specific nutrient−sensing mechanism or a broader cellular stress response. Future studies incorporating metabolic monitors -such as LC3−II accumulation, phospho−mTOR/AMPK levels, or ROS quantification- will be essential to distinguish direct nutrient−sensing effects from generalized stress responses and to clarify how metabolic cues intersect with CD3−dependent signaling in macrophages.

Regarding intrinsic phagocytic capability, CD3+ RAW cells retained this function to a degree comparable to their CD3- counterparts. These findings do not definitively establish a direct role for CD3 in phagocytosis; rather, they may suggest that CD3+ RAW cells can phagocytose, even though they are considered a non-classical subpopulation. Further studies are necessary to investigate if, in an infectious context, these cells retain the ability to degrade and present antigens, as in classical phagocytic cells.

TCR/CD3 complex interaction with FcγR triggers secondary signaling that synergizes with the TCR/CD3 complex in T cells (22). In particular, this synergistic effect during the activation of naïve CD4+ T cells is relevant: non-activated CD4+ T cells do not express FcRs, whereas once activated, they express FcγRIIIa. The engagement of FcγRIIIa by immune complexes provides a crucial costimulatory signal that promotes the differentiation of CD4+ T cells into effector cells (23). Moreover, CD3 ligation in combination with Fcγ receptor co-stimulation induced a rapid and sustained IL-2 response (10).

This study demonstrates that anti-CD3/IgG stimulation induces a distinct inflammatory cytokine profile over time, initially marked by IFN-γ production and subsequently by sustained TNF release, indicating persistent inflammatory signaling. Furthermore, a transient peak in IL-6 was observed. These temporal cytokine expression patterns align with responses observed in chimeric antigen receptor macrophages (CAR-M) and M2 macrophages, which are increasingly recognized as next-generation cellular therapies that enhance phagocytic capacity and pro-inflammatory cytokine expression, including IL-1β, IL-6, and TNF (24, 25).

Typically, this crosstalk is essential for preventing cellular anergy, particularly in T lymphocytes. Our findings indicate that in the RAW cell model, the use of anti-CD3/IgG enhances cytokine production and accelerates the early activation dynamics of NFAT-1, IKK-α, and c-Jun, while NF-κB activation is consequently delayed. This temporal pattern closely resembles the canonical TCR signaling observed in T lymphocytes, suggesting that RAW cells can partially emulate TCR-linked signaling cascades. The delayed activation of NF-κB plausibly indicates macrophage-specific modulation within the TCR/CD3 signaling axis. Indeed, T cells maintain a temporal balance between NFAT and NF-κB (26). However, in our stimulation strategy should be interpreted with caution because Fcγ receptors are potent activators of macrophages and can independently induce an inflammatory response. Therefore, the phenotype observed here cannot be unequivocally attributed to CD3 signaling alone, it may result from CD3 activation, FcγR-II/III engagement, or a true crosstalk between both receptors, as has been described in other myeloid cell contexts. Thus, although our data support a functional role for CD3 in promoting a proinflammatory phenotype in RAW macrophages, we recognize that FcγR engagement and its integration with CD3-derived signals are likely to contribute to the magnitude and kinetics of the observed response.

Previous evidence has shown that NFAT-1 is predominantly regulated in RAW cells in response to RANKL stimulation, underscoring its critical role in the synthesis and secretion of inflammatory cytokines (27). Furthermore, experimental models involving NFAT-1 ablation have demonstrated the induction of GATA2 and STAT6 activation as alternative pathways, without suppressing the expression of TNF-α and IL-6; however, the expression of certain cytokines such as IL-13, which is associated with macrophage differentiation into the M2 phenotype, is impaired, indicating that this pathway is vital for maintaining sustained pro-inflammatory activation (28). Activation via crosstalk between CD3 and IgG may preferentially induce signaling by activating NFAT-1, while concurrently inhibiting the expression of GATA-3 and MAFB—transcription factors associated with an anti-inflammatory profile. Notably, MAFB is linked to anti-inflammatory programs and the identity of tissue-resident macrophages (29–31). GATA-3, well recognized for its role in Th2 cell differentiation, also participates in macrophage polarization and the modulation of inflammatory signaling (32). Collectively, these transcription factors—NFAT-1, GATA2, STAT6, GATA-3, and MAFB—constitute an initial molecular screening toolkit for evaluating macrophage responses following anti-CD3 stimulation. Their expression patterns facilitate the inference of the directionality of activation profiles in RAW cells.

IRF-1 is a pivotal transcription factor that directs proinflammatory and antimicrobial responses in macrophages, particularly in response to IFN-γ and toll-like receptor signaling pathways (33, 34). Our transcriptional analysis provided additional evidence of proinflammatory programming in RAW cells. Notably, IRF-1 expression was elevated early following anti-CD3 stimulation, aligning with prior reports indicating that anti-CD3 stimulation induces IRF-1 expression in T cells (35). These findings underscore the functional significance of IRF-1 in the proinflammatory programming of macrophages after CD3 engagement.

Our data indicate that FcRs, which are non-PRRs, have the capacity to induce transcriptional reprogramming in RAW cells. It is hypothesized that crosstalk signaling enhances glycolysis in CD3+ RAW cells through the kinases Syk and PI3K, analogous to previous findings in dendritic cells involving an IgA-IC pathway. This process consequently augments fatty acid synthesis and endoplasmic reticulum expansion, resulting in elevated gene translation (36). FcR signaling occurs via an immunoreceptor tyrosine-based activation motif, a conserved signaling module predominantly utilized in leukocytes (37). Notably, stimulation through cross-talk, which activates FcRs signaling via ITAMs, has been observed to upregulate proinflammatory cytokine production in a manner comparable to that of IgA-IC in dendritic cells or CD3-FcRs (37, 38).

Collectively, these data support a model wherein CD3 expression in RAW cells is functionally associated with macrophage activation and inflammatory signaling. Instead of representing a remnant of aberrant transcription, CD3 appears to contribute to mediate an inflammatory program involving *TCR-like* signaling pathways. Importantly, future loss-of-function studies, including knockdown or siRNA-mediated silencing of CD3, will be required to establish whether CD3 plays a causal role and to strengthen the use of RAW cells as a model for studying these unconventional myeloid populations. An important question for future studies is whether non-immortalized human myeloid cells also exhibit increased CD3 expression under nutrient-deprivation conditions, as observed in various infectious and inflammatory diseases, including tuberculosis, hepatitis, pneumonitis, and cancer, where CD3+ monocytes/macrophages have been documented.

Although our study identifies important associations, it exhibits several limitations impede definitive conclusions regarding causality. First, the absence of CD3 loss-of-function experiments prevents us from establishing a causal role for CD3 in the observed inflammatory phenotype. Second, our stimulation strategy did not allow discrimination between signaling mediated by CD3 alone, FcγR-II/III alone, or crosstalk. And third, additional functional studies such as antigen presentation, ROS production, metabolic profiling, and receptor-specific inhibition would further define the biological significance of CD3-associated macrophage activation.

## 5 Conclusions

Our findings demonstrate that RAW cells express multiple molecules classically associated with T cells, including CD3, TCR, CD4, and CD8, and exhibit functional responses following CD3 engagement. Upon anti-CD3 stimulation, CD3+ RAW cells displayed phagocytic activity and a rapid, proinflammatory cytokine profile, accompanied by early activation of signaling pathways including NFAT, C-Jun, and IKK, and delayed NF-κB activation. The observed modulation of CD3 expression in response to nutrient stress suggests dynamic regulation associated with environmental factors. Together, these results underscore the utility value of RAW cells as a model for studying noncanonical CD3/TCR signaling in macrophages and emphasize the significance of this pathway in influencing innate immune responses.

## Data Availability

The original contributions presented in the study are included in the article/**Supplementary Material**. Further inquiries can be directed to the corresponding author.

## References

[1] Puellmann K Kaminski WE Vogel M Nebe CT Schroeder J Wolf H . A variable immunoreceptor in a subpopulation of human neutrophils. Proc Natl Acad Sci. (2006) 103:14441–6. doi: 10.1073/pnas.0603406103 16983085 PMC1599981

[2] Fuchs T Hahn M Riabov V Yin S Kzhyshkowska J Busch S . A combinatorial αβ T cell receptor expressed by macrophages in the tumor microenvironment. Immunobiology. (2017) 222:39–44. doi: 10.1016/j.imbio.2015.09.022 26494401

[3] Fuchs T Puellmann K Emmert A Fleig J Oniga S Laird R . The macrophage-TCRαβ is a cholesterol-responsive combinatorial immune receptor and implicated in atherosclerosis. Biochem Biophys Res Commun. (2015) 456:59–65. doi: 10.1016/j.bbrc.2014.11.034 25446098

[4] Beham AW Puellmann K Laird R Fuchs T Streich R Breysach C . A TNF-regulated recombinatorial macrophage immune receptor implicated in granuloma formation in tuberculosis. PloS Pathog. (2011) 7:e1002375. doi: 10.1371/journal.ppat.1002375 22114556 PMC3219713

[5] Chavez-Galan L Vesin D Blaser G Uysal H Benmerzoug S Rose S . Myeloid cell TNFR1 signaling dependent liver injury and inflammation upon BCG infection. Sci Rep. (2019) 9:5297. doi: 10.1038/s41598-019-41629-9 30923339 PMC6438980

[6] Rodriguez-Cruz A Vesin D Ramon-Luing L Zuñiga J Quesniaux VFJ Ryffel B . CD3+ macrophages deliver proinflammatory cytokines by a CD3- and transmembrane TNF-dependent pathway and are increased at the BCG-infection site. Front Immunol. (2019) 10. doi: 10.3389/fimmu.2019.02550 31787969 PMC6855269

[7] Ocaña-Guzmán R Téllez-Navarrete NA Ramón-Luing LA Herrera I Ita MD Carrillo-Alduenda J-L . Leukocytes from patients with drug‐sensitive and multidrug‐resistant tuberculosis exhibit distinctive profiles of chemokine receptor expression and migration capacity. J Immunol Res. (2021) 21:6654220. doi: 10.1155/2021/6654220 PMC8084684 33977111

[8] Ramon-Luing LA Carranza C Téllez-Navarrete NA Medina-Quero K Gonzalez Y Torres M . Mycobacterium tuberculosis H37Rv strain increases the frequency of CD3+TCR+ macrophages and affects their phenotype, but not their migration ability. Int J Mol Sci. (2021) 23(1):329 doi: 10.3390/ijms23010329 35008755 PMC8745617

[9] Ocaña-Guzmán R Ramón-Luing LA Rodríguez-Alvarado M Voss T-D Fuchs T Chavez-Galan L . Murine RAW macrophages are a suitable model to study the CD3 signaling in myeloid cells. Cells. (2022) 11(10):1635. doi: 10.3390/cells11101635 35626672 PMC9139304

[10] Antonopoulou T Kanakousaki E Dimitropoulos C Manidakis N Athanassakis I . Aberrant expression of T cell receptors in monocyte/macrophage RAW 264.7 cells: FCγRII/III compensates the need for CD3. Mol Immunol. (2023) 157:167–75. doi: 10.1016/j.molimm.2023.03.022 37028131

[11] Okada K Mori D Makii Y Nakamoto H Murahashi Y Yano F . Hypoxia-inducible factor-1 alpha maintains mouse articular cartilage through suppression of NF-κB signaling. Sci Rep. (2020) 10:5425. doi: 10.1038/s41598-020-62463-4 32214220 PMC7096515

[12] Taciak B Białasek M Braniewska A Sas Z Sawicka P Kiraga Ł . Evaluation of phenotypic and functional stability of RAW 264.7 cell line through serial passages. PloS One. (2018) 13:e0198943. doi: 10.1371/journal.pone.0198943 29889899 PMC5995401

[13] Zhen A Krutzik SR Levin BR Kasparian S Zack JA Kitchen SG . CD4 ligation on human blood monocytes triggers macrophage differentiation and enhances HIV infection. J Virol. (2014) 88:9934–46. doi: 10.1128/JVI.00616-14 24942581 PMC4136363

[14] Geisler C . TCR trafficking in resting and stimulated T cells. Crit Rev Immunol. (2004) 24:67–86. doi: 10.1615/critrevimmunol.v24.i1.30 14995914

[15] Davern M Donlon NE O’Connell F Gaughan C O’Donovan C McGrath J . Nutrient deprivation and hypoxia alter T cell immune checkpoint expression: potential impact for immunotherapy. J Cancer Res Clin Oncol. (2023) 149:5377–95. doi: 10.1007/s00432-022-04440-0 36445478 PMC10349772

[16] Rodriguez PC Zea AH Culotta KS Zabaleta J Ochoa JB Ochoa AC . Regulation of T cell receptor CD3ζ chain expression byl-arginine *. J Biol Chem. (2002) 277:21123–9. doi: 10.1074/jbc.M110675200 11950832

[17] Mejlvang J Olsvik H Svenning S Bruun J-A Abudu YP Larsen KB . Starvation induces rapid degradation of selective autophagy receptors by endosomal microautophagy. J Cell Biol. (2018) 217:3640–55. doi: 10.1083/jcb.201711002 30018090 PMC6168274

[18] Yang Y Sun L Chen Z Liu W Xu Q Liu F . The immune-metabolic crosstalk between CD3+C1q+TAM and CD8+T cells associated with relapse-free survival in HCC. Front Immunol. (2023) 14. doi: 10.3389/fimmu.2023.1033497 36845133 PMC9948089

[19] Le Tonqueze O Kollu S Lee S Al-Salah M Truesdell SS Vasudevan S . Regulation of monocyte induced cell migration by the RNA binding protein, FXR1. Cell Cycle. (2016) 15:1874–82. doi: 10.1080/15384101.2016.1189040 27229378 PMC4968908

[20] Missiaen R Lesner NP Simon MC . HIF: a master regulator of nutrient availability and metabolic cross‐talk in the tumor microenvironment. EMBO J. (2023) 42:EMBJ2022112067. doi: 10.15252/embj.2022112067 36808622 PMC10015374

[21] Gameiro PA Struhl K . Nutrient deprivation elicits a transcriptional and translational inflammatory response coupled to decreased protein synthesis. Cell Rep. (2018) 24:1415–24. doi: 10.1016/j.celrep.2018.07.021 30089253 PMC6419098

[22] Dadas O Allen JD Buchan SL Kim J Chan HTC Mockridge CI . Fcγ receptor binding is required for maximal immunostimulation by CD70-Fc. Front Immunol. (2023) 14. doi: 10.3389/fimmu.2023.1252274 37965342 PMC10641686

[23] Chauhan AK . Human CD4+ T-cells: A role for low-affinity Fc receptors. Front Immunol. (2016) 7. doi: 10.3389/fimmu.2016.00215 27313579 PMC4887501

[24] Hong J Lee S Kim Y Kang CK Park WB Shin HM . Optimization of THP-1-CAR monocytes utilizing CD32a signaling phagocytosis for antigen-specific T cell activation. Sci Rep. (2026) 16:8175. doi: 10.1038/s41598-026-39406-6 41663634 PMC12963538

[25] Vogelpoel LTC Hansen IS Rispens T Muller FJM van Capel TMM Turina MC . Fc gamma receptor-TLR cross-talk elicits pro-inflammatory cytokine production by human M2 macrophages. Nat Commun. (2014) 5:5444. doi: 10.1038/ncomms6444 25392121 PMC4243215

[26] Huang W Lin W Chen B Zhang J Gao P Fan Y . NFAT and NF-κB dynamically co-regulate TCR and CAR signaling responses in human T cells. Cell Rep. (2023) 42:112663. doi: 10.1016/j.celrep.2023.112663 37347664

[27] Sun Y Tao Y Geng Z Zheng F Wang Y Wang Y . The activation of CaN/NFAT signaling pathway in macrophages aggravated Lactobacillus casei cell wall extract-induced Kawasaki disease vasculitis. Cytokine. (2023) 169:156304. doi: 10.1016/j.cyto.2023.156304 37487381

[28] Russo R Mallia S Zito F Lampiasi N . Gene expression profiling of NFATc1-knockdown in RAW 264.7 cells: An alternative pathway for macrophage differentiation. Cells. (2019) 8:131. doi: 10.3390/cells8020131 30736420 PMC6406727

[29] Vanneste D Peng W Liu Z Hamaïdia M Rocca L Abinet J . MafB is a conserved transcriptional regulator of macrophage development and functional identity across tissues and species. Immunity. (2026) 59:559–576.e12. doi: 10.1016/j.immuni.2026.01.012 41759510 PMC7618887

[30] Kim H . The transcription factor MafB promotes anti-inflammatory M2 polarization and cholesterol efflux in macrophages. Sci Rep. (2017) 7:7591. doi: 10.1038/s41598-017-07381-8 28790455 PMC5548719

[31] Hikichi H Seto S Wakabayashi K Hijikata M Keicho N . Transcription factor MAFB controls type I and II interferon response-mediated host immunity in Mycobacterium tuberculosis-infected macrophages. Front Microbiol. (2022) 13. doi: 10.3389/fmicb.2022.962306 36406405 PMC9670303

[32] Lawrence T Natoli G . Transcriptional regulation of macrophage polarization: enabling diversity with identity. Nat Rev Immunol. (2011) 11:750–61. doi: 10.1038/nri3088 22025054

[33] Wu D Pan P Su X Zhang L Qin Q Tan H . Interferon regulatory factor-1 mediates alveolar macrophage pyroptosis during LPS-induced acute lung injury in mice. Shock. (2016) 46:329. doi: 10.1097/SHK.0000000000000595 26939040 PMC4978602

[34] Rosain J Neehus A-L Manry J Yang R Pen JL Daher W . Human IRF1 governs macrophagic IFN-γ immunity to mycobacteria. Cell. (2023) 186:621–645.e33. doi: 10.1016/j.cell.2022.12.038 36736301 PMC9907019

[35] Sgarbanti M Marsili G Remoli AL Stellacci E Mai A Rotili D . IκB kinase ϵ targets interferon regulatory factor 1 in activated T lymphocytes. Mol Cell Biol. (2014) 34:1054–65. doi: 10.1128/MCB.01161-13 24396068 PMC3958032

[36] Hansen IS Krabbendam L Bernink JH Loayza-Puch F Hoepel W van Burgsteden JA . FcαRI co-stimulation converts human intestinal CD103+ dendritic cells into pro-inflammatory cells through glycolytic reprogramming. Nat Commun. (2018) 9:863. doi: 10.1038/s41467-018-03318-5 29491406 PMC5830413

[37] Humphrey MB Lanier LL Nakamura MC . Role of ITAM-containing adapter proteins and their receptors in the immune system and bone. Immunol Rev. (2005) 208:50–65. doi: 10.1111/j.0105-2896.2005.00325.x 16313340

[38] Guy CS Vignali DAA . Organization of proximal signal initiation at the TCR:CD3 complex. Immunol Rev. (2009) 232:7–21. doi: 10.1111/j.1600-065X.2009.00843.x 19909352 PMC2845712

