# Supplementary material for: Nutrient deprivation increases CD3 expression in RAW cells and augments the CD3-induced proinflammatory profile, associated with NFAT and IRF-1
Source: Front Immunol. 2026 Jul 21;17:1857007. doi: 10.3389/fimmu.2026.1857007 (PMC13433164; doi:10.3389/fimmu.2026.1857007)
Supplement: Supplementary file 1 [file DataSheet1.docx]

Supplementary Material

**Supplementary Figure 1. Analysis of signaling pathway activation in RAW cells following anti-CD3/IgG2a stimulation.** RAW 264.7 cells were stimulated under unstimulated conditions (RPMI) as a basal control, stimulated with anti-CD3 plus IgG2a (anti-CD3/IgG), treated with isotype control antibodies (isotype control), or stimulated with positive controls such as lipopolysaccharide (LPS) or PMA plus ionomycin (PMA/IO). Whole-cell lysates were collected at early time points for Western blot analysis. Representative immunoblots of NFAT-1, pC-Jun, NF-κB, IKK-α, and STAT-1 are shown at 5 and 10 min in (A), and at 20 and 60 min in (B). β-Actin was used as a loading control. Representative membranes of n = 6.

**Supplementary Figure 2. Anti-CD3/IgG2a activates signaling pathways involving NF-kB, NFAT-1 and C-Jun.** Representative blots of RAW cells protein extracts, obtained from cultures under different experimental conditions: Unstimulated as control conditions (RPMI), stimulated with anti-CD3 (anti-CD3), or stimulated with anti-CD3 + IgG2a (anti-CD3/IgG). Signaling proteins such as IKKa (B), IK-B (C) and NF-kB (D) were examined. GAPDH was used as loading control. Data shown were represented as mean ± SD (n=3).
